# Recent Origin and Cultural Reversion of a Hunter–Gatherer Group

**DOI:** 10.1371/journal.pbio.0030071

**Published:** 2005-02-22

**Authors:** Hiroki Oota, Brigitte Pakendorf, Gunter Weiss, Arndt von Haeseler, Surin Pookajorn, Wannapa Settheetham-Ishida, Danai Tiwawech, Takafumi Ishida, Mark Stoneking

**Affiliations:** **1**Max Planck Institute for Evolutionary AnthropologyLeipzigGermany; **2**WE Informatik, Heinrich-Heine-UniversitätDüsseldorfGermany; **3**Neumann-Institute for Computing, Forschungszentrum JülichDüsseldorfGermany; **4**Faculty of Archaeology, Silpakorn UniversityBangkokThailand; **5**Faculty of Medicine, Khon Kaen UniversityKhon KaenThailand; **6**National Cancer InstituteBangkokThailand; **7**Department of Biological Sciences, School of ScienceUniversity of TokyoJapan; Massey UniversityNew Zealand

## Abstract

Contemporary hunter–gatherer groups are often thought to serve as models of an ancient lifestyle that was typical of human populations prior to the development of agriculture. Patterns of genetic variation in hunter–gatherer groups such as the !Kung and African Pygmies are consistent with this view, as they exhibit low genetic diversity coupled with high frequencies of divergent mtDNA types not found in surrounding agricultural groups, suggesting long-term isolation and small population sizes. We report here genetic evidence concerning the origins of the Mlabri, an enigmatic hunter–gatherer group from northern Thailand. The Mlabri have no mtDNA diversity, and the genetic diversity at Y-chromosome and autosomal loci are also extraordinarily reduced in the Mlabri. Genetic, linguistic, and cultural data all suggest that the Mlabri were recently founded, 500–800 y ago, from a very small number of individuals. Moreover, the Mlabri appear to have originated from an agricultural group and then adopted a hunting–gathering subsistence mode. This example of cultural reversion from agriculture to a hunting–gathering lifestyle indicates that contemporary hunter–gatherer groups do not necessarily reflect a pre-agricultural lifestyle.

## Abstract 2

Genes, language and culture reveal that the Mlabri reverted from an agricultural to a hunter-gatherer lifestyle, suggesting that hunter-gatherer groups might not always represent the pre- agricultural lifestyle of humans

## Introduction

The Mlabri are an enigmatic group of about 300 people who nowadays range across the Nan, Phrae, and Phayao provinces of north and northeastern Thailand and the Sayaburi province of western Laos [1,2]. Their traditional lifestyle is to move frequently through the dense forests of the high mountains, building temporary structures of bamboo sticks thatched with banana leaves, which they occupy for a few days, until the leaves turn yellow (thus accounting for their traditional Thai name, Phi Tong Luang, which means “spirit of the yellow leaves”). First contacted by Europeans in 1936 [3], they are unique among the hill tribes of northern Thailand in that, until recently, they subsisted by hunting and gathering combined with occasional barter trade with villagers.

The origins of the Mlabri are controversial. Some investigators have assumed that there is a direct connection between the Mlabri and the ancient Hoabinhian hunting–gathering culture of Southeast Asia [1]. However, a limited investigation of blood group variation [4] raised the possibility that the Mlabri originated via a founder event from an agricultural group, and preliminary linguistic analyses support this idea. The Mlabri language seems lexically most closely related to Khmu and Tin, two languages of the Khmuic branch of the Mon-Khmer sub-family of Austro-Asiatic languages, both of which are spoken in agricultural highland villages [5]. The cluster of dialects jointly referred to as Tin, or Mal/Prai, [6] is spoken in the Thailand–Laos border region that the Mlabri also occupy, whereas Khmu is spoken over a much wider area [7]. The grammar of Mlabri additionally has features that deviate markedly from typical Mon-Khmer, suggesting that Mlabri developed as a result of contact between speakers of a Khmuic language and speakers of a quite different language of unknown affiliation [2,8].

We report here the results of an investigation of genetic diversity in the Mlabri, to see whether patterns of genetic variation might provide further insights into the question of an agricultural versus hunting–gathering origin for the Mlabri. The rationale for using genetic analyses to investigate this question is that previous work has shown that hunter–gatherer groups typically differ from their agricultural neighbors in having reduced genetic diversity and high frequencies of unique mtDNA types [9,10,11,12,13,14], so we might expect a similar pattern if the Mlabri have always been hunter–gatherers. The genetic results, combined with linguistic and cultural evidence, suggest that the most probable explanation for the origin of the Mlabri is an extreme founder event from an agricultural group, followed by adoption of a hunting–gathering lifestyle.

## Results/Discussion

### Genetic Analyses: mtDNA Diversity

We analyzed 360 bp of the first hypervariable segment (HV1) of the mtDNA control region in 58 Mlabri; surprisingly, all of the sequences were identical, with the following differences from the reference sequence [15]: 16140C, 16189C, and 16266A, as well as the common Asian 9-bp deletion in the intergenic region between the cytochrome oxidase subunit II and lysine tRNA genes [16]. No other human population has been found to lack mtDNA HV1 variation, and mtDNA HV1 variation in six other hill tribes (all agricultural groups) from the same region of Thailand was significantly higher (Figure 1; Table 1).

**Figure 1:**
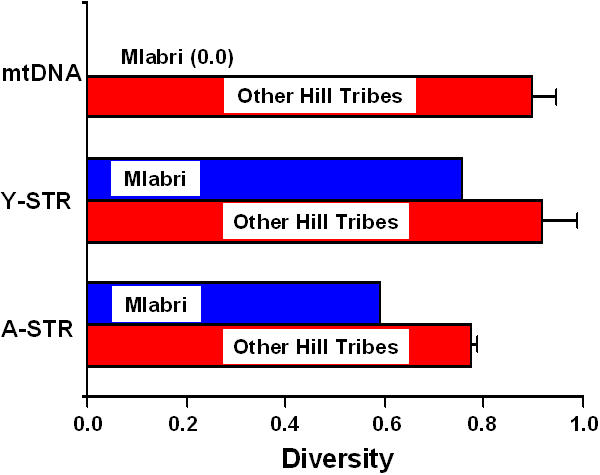
Genetic Diversity in the Mlabri and Other Hill Tribes Genetic diversity based on mtDNA HV1 sequences, Y-STR haplotypes, and autosomal STR (A-STR) genotypes in the Mlabri, compared to the average genetic diversity for six other hill tribes. The haplotype diversity is indicated for the mtDNA and Y-STR data, while the average heterozygosity is indicated for the autosomal STR loci.

**Table 1:**
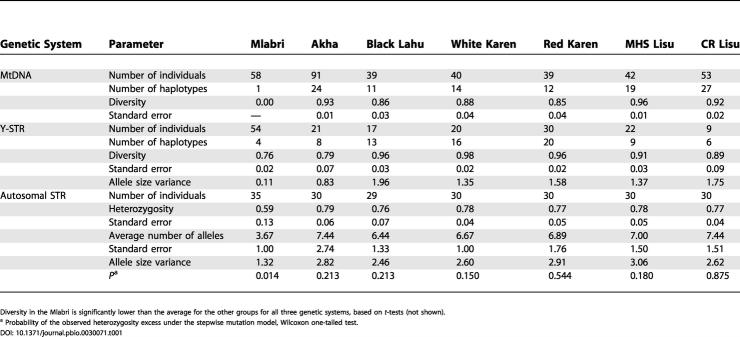
Genetic Diversity Parameters Based on mtDNA HV1 Sequences, Y-STR Haplotypes, and Autosomal STR Genotypes for the Mlabri and the Six Other Hill Tribes

Diversity in the Mlabri is significantly lower than the average for the other groups for all three genetic systems, based on *t*-tests (not shown)

^a^ Probability of the observed heterozygosity excess under the stepwise mutation model, Wilcoxon one-tailed test

### Y-Chromosome Diversity

We analyzed nine short tandem repeat (STR) loci on the Y chromosome in 54 Mlabri, and again found significantly reduced variation in the Mlabri compared to the other six hill tribes (Figure 1; Table 1). The Mlabri had just four Y-chromosome STR (Y-STR) haplotypes, two of which differed by a single repeat at a single locus from one each of the other two haplotypes (Table 2). The Y-STR haplotype diversity in the Mlabri is again lower than that reported for any other human population [17,18]; the Akha, one of the six other hill tribes, also exhibited very low Y-STR diversity (Table 1). The average variance in the allele size distribution at the nine Y-STR loci shows an even greater contrast between the Mlabri and the other hill tribes: the average variance was 0.11 for the Mlabri, versus an average of 1.45 for the other six hill tribes.

**Table 2:**
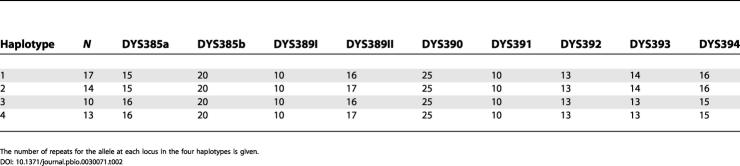
Y-STR Haplotypes in the Mlabri

The number of repeats for the allele at each locus in the four haplotypes is given

### Autosomal DNA Diversity

We analyzed nine autosomal STR loci in the Mlabri and the other six hill tribes, and again found significantly reduced variation in the Mlabri (Figure 1; Table 1). The genotype frequencies did not deviate significantly from Hardy–Weinberg expectations for any locus in the Mlabri; however, even though these nine STR loci are on different chromosomes and hence unlinked, eight pairs of loci exhibited significant linkage disequilibrium (LD) (*p* < 0.05; Figure 2), as measured by a likelihood ratio test [19]. This is significantly more (*p* < 0.01) than the 1.8 pairs expected by chance (out of 36 pairwise comparisons) to exhibit this level of LD. For each of the six agricultural hill tribes, the number of pairs of loci exhibiting significant LD was within expectations (Figure 2). Moreover, the *p*-value of the likelihood ratio test is a measure of the strength of the association between two loci [19]; the average *p*-value was 0.20 for the Mlabri, versus 0.31–0.55 for the other six hill tribes, indicating that overall associations between these unlinked loci were stronger in the Mlabri than in the other hill tribes. However, the sample size for the Mlabri for the autosomal STR analyses was larger than the sample size for the other hill tribes (*n* = 35 for the Mlabri, versus *n* = 29–30 for the others), so it is possible that the lower average *p*-value for the Mlabri reflects more statistical power due to a larger sample size and not more LD. To test this, we sampled 30 Mlabri at random and redid the LD analysis; the conclusions did not change, indicating that the lower average *p-*value for the Mlabri does reflect more LD in the Mlabri.

**Figure 2:**
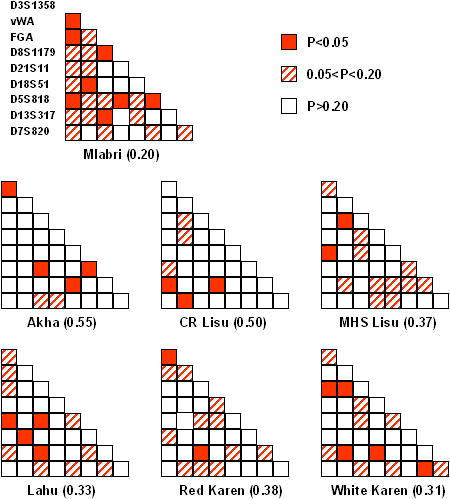
Associations amongst Unlinked Autosomal STR Loci in the Mlabri and the Other Hill Tribes Probability values of the likelihood ratio test of association versus no association for nine unlinked autosomal STR loci in the Mlabri and six other hill tribes (average probability over the 36 pairs of loci in parentheses).

One explanation for the reduced diversity at mtDNA, Y-STR loci, and autosomal STR loci, and the significant number of pairs of unlinked autosomal STR loci in LD, is a severe reduction in population size in the Mlabri. Following such an event, the number of alleles is reduced more than the heterozygosity, leading to an excess of observed heterozygosity compared to that expected for the observed number of alleles under mutation–drift equilibrium [20]. We therefore compared the observed and expected heterozygosity (at mutation–drift equilibrium, conditioned on the observed number of alleles) for the autosomal STR loci in the Mlabri and the six other hill tribes, under a stepwise mutation model. Only the Mlabri exhibited a significant excess of observed heterozygosity (Table 1). Although more complicated scenarios are possible, the simplest explanation is that the Mlabri (but not the other hill tribes) have undergone a severe reduction in population size, as also indicated by the mtDNA and Y-STR haplotype data, and as also suggested by a previous study of blood group variation [4].

### Population Size Reduction in the Mlabri

Assuming that there was a reduction in population size in the Mlabri that set the mtDNA and Y-chromosome diversity near or equal to zero, the coalescence times for the Mlabri mtDNA and Y-STR haplotypes provide an upper estimate as to when the population reduction occurred. We therefore applied Bayesian-based coalescence analysis [21] to the mtDNA sequences and the Y-STR haplotypes from the Mlabri and the other six hill tribes. For the six agricultural hill tribes, the resulting estimates of coalescence time are broadly distributed (Figure 3), indicating little information in the data (except for the Akha, who do show a pronounced peak in the posterior probability distribution for the Y-STR data, in accordance with their lower Y-STR haplotype diversity). By contrast, the estimates of coalescence time for the Mlabri show a sharp peak (Figure 3), with a median time of 770 y (approximate 95% credible interval 250–4,270 y) for the mtDNA sequences and 490 y (approximate 95% credible interval 170–1,290 y) for the Y-STR haplotypes.

**Figure 3:**
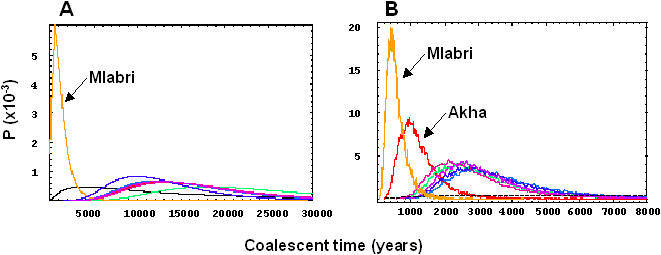
Time to the Most Recent Common Ancestor for mtDNA and Y-STR Types for the Mlabri and the Other Hill Tribes Posterior probability distribution of the time back to the most recent common ancestor for the mtDNA (A) and Y-STR haplotype (B) data for the Mlabri and six other hill tribes.

Both the mtDNA and the Y-STR data therefore indicate that the Mlabri underwent a substantial reduction in population size about 500–800 y ago (and not more than about 1,300 y ago, if the mtDNA and Y-chromosome data reflect the same event). There are two possible scenarios: (1) a bottleneck, in which the Mlabri were reduced from a formerly large population to a much smaller population size, which then increased to the current level of about 300 individuals; or (2) a founder event, in which the Mlabri began as a very small number of individuals, became isolated, and then increased over time to their present size. Similar reductions in genetic diversity are predicted under either scenario, so the genetic data cannot distinguish between these. But some information can be obtained by considering the magnitude of the reduction in population size needed to completely eliminate mtDNA diversity in the Mlabri.

The amount of population size reduction needed to completely eliminate mtDNA diversity in the Mlabri depends on how much mtDNA diversity was present prior to the size reduction. We assumed that the ancestral Mlabri population would have the same mtDNA diversity as one of the other hill tribes and then estimated the amount of population size reduction needed to completely eliminate mtDNA diversity by resampling with replacement various numbers of mtDNA types from the ancestral (pre-bottleneck) population. For example, we started with an ancestral population with the same distribution of mtDNA types as the Akha. We then sampled two mtDNA types (with replacement) from this ancestral population, repeated this procedure 1,000 times, and found that 243 out of the 1,000 resamples of size two had no mtDNA diversity; thus, the probability is 0.243 that a reduction to just two individuals would eliminate mtDNA diversity in an ancestral population that started with the same mtDNA diversity as the Akha. We then repeated this procedure, sampling three mtDNA types (with replacement), and obtained a probability of 0.007 that there would be no mtDNA diversity following a reduction to three individuals. Therefore, if the Mlabri were derived from a population with the same mtDNA diversity as the Akha, the population had to be reduced to not more than two unrelated females, in order to completely eliminate mtDNA diversity.

This resampling analysis was carried out six times, with the putative ancestral mtDNA diversity corresponding to each of the six hill tribes. The results of this analysis were that for five of the ancestral populations, resampling three (or more) individuals gave a probability of no mtDNA diversity of less than 0.05; for the remaining ancestral population (which had the same starting mtDNA diversity as the Red Karen), resampling four (or more) individuals gave a probability of no mtDNA diversity of less than 0.05.

We also carried out a similar analysis for the Y-STR types in the Mlabri. Here we again assumed an ancestral population with the same Y-STR haplotype diversity as one of the other hill tribes, then determined the maximum number of individuals that could be sampled at random that would have not more than two Y-STR types (since the four Y-STR types in the Mlabri consist of two pairs that differ by a single-step mutation at a single locus). The results of this analysis were that at most 3–6 individuals (depending on which hill tribe the ancestral population resembled most in terms of Y-STR diversity) could have been present after the size reduction, otherwise, with greater than 95% probability, more than two Y-STR types would have been retained.

A critical assumption is the amount of genetic diversity present in the ancestral Mlabri population prior to the size reduction. The estimates used in the above analysis are based on agricultural populations, which in general have more mtDNA diversity than hunter–gatherer populations. We therefore also constructed putative ancestral populations with frequency distributions of mtDNA types identical to those found in the !Kung and in African Pygmies [22]; the results of the resampling analysis were the same.

Another assumption of this analysis is that the event that led to the population size reduction completely eliminated the mtDNA diversity. Alternatively, some mtDNA diversity may have been present after the population size reduction, but was subsequently lost because of drift. Loss of mtDNA diversity due to subsequent drift is not likely if there was a single event reducing the Mlabri population size that was followed by population growth, since mtDNA diversity is retained in growing populations [23]. However, if the reduction in size occurred over several generations, then it may not have been as dramatic a bottleneck as the resampling analysis implies.

To investigate this further, we employed a Bayesian approach, following the procedure previously used to estimate the number of founders for the Maoris [24] but allowing for new mutations, to estimate the number of founders for the Mlabri, assuming various time periods since the founding event. The results (Figure 4) indicate that the most probable number of founders is one over all time periods; however, for longer time periods since the founding event, there is decreasing information on the number of founders from the observation of no mtDNA diversity in the Mlabri. As expected, the longer the time since the founding event (i.e., the slower the population growth rate), the greater the influence of drift in eliminating diversity that might have been present in the founding population. Nevertheless, given that the coalescent analyses indicate an upper date for the origin of the Mlabri of about 1,000 y ago, the lack of mtDNA diversity in the Mlabri is most consistent with a very small founding population size, perhaps even only one female lineage.

**Figure 4:**
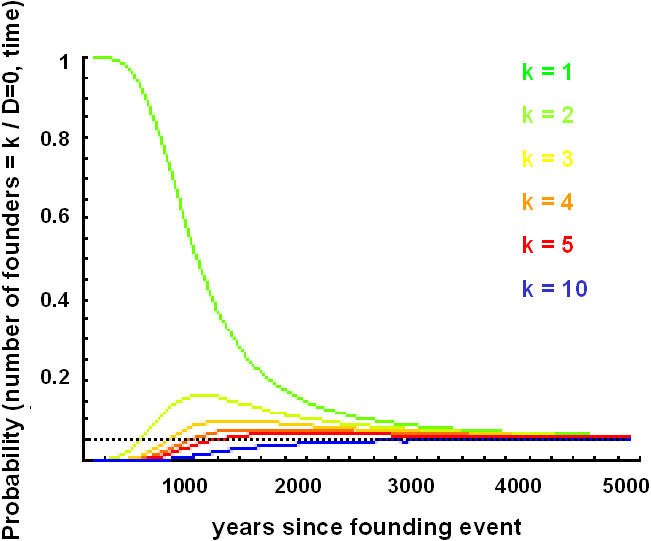
Number of Founding Individuals in the Mlabri, Given No mtDNA Diversity Posterior probability distribution for the number of founding individuals *(k),* conditioned on the observation of no diversity in a sample of 58 mtDNA sequences and the time since the founding event. The prior probability is indicated by the dashed black line.

### Origin of the Mlabri

The group that gave rise to the founder event that established the Mlabri could have been either a hunter–gatherer group, in which case the Mlabri maintained their hunting–gathering lifestyle from before, or an agricultural group, in which case the Mlabri subsequently adopted their current hunting–gathering lifestyle. While the genetic data cannot unequivocally distinguish between these two possibilities, they do suggest the latter. Other hunter–gatherer groups typically share few, if any, mtDNA types with neighboring agricultural groups, consistent with long-term isolation of the hunter–gatherer groups. For example, !Kung, African Pygmies, Andamanese Islanders, and south Indian hunter–gatherer groups can readily be distinguished from nearby agricultural groups on the basis of their mtDNA sequences [9,13,14,25]. By contrast, the Mlabri mtDNA sequence has been reported in other, agricultural hill tribes [26,27], and identical or closely related sequences have also been reported from Southeast Asia and China [9,28,29]. Similarly, the Mlabri Y-STR haplotypes are identical or closely related (differing by a single-step mutation at one locus) to Y-STR haplotypes found in Southeast Asia and Oceania [30,31]. Also, the Mlabri do not exhibit any alleles at the nine autosomal STR loci that are not found in the agricultural hill tribes.

The widespread sharing of mtDNA, Y-STR, and autosomal STR alleles between the Mlabri and agricultural groups in Southeast Asia are not expected if the Mlabri have always been hunter–gatherers. Instead, the genetic data suggest that the Mlabri are derived from an agricultural group. Moreover, the Mlabri vocabulary and folklore also give some evidence of ancient familiarity with agriculture coexisting with hunting and gathering (J. Rischel, personal communication). While preliminary in nature, the available linguistic evidence suggests that the present-day Mlabri language arose after some speakers of a Khmuic language, most likely Tin, became isolated and subsequently experienced intensive contact with speakers of some other, presently unknown language [2,8]. Just how long ago the Mlabri and Tin languages diverged cannot be determined, but it has been suggested that Tin branched from Khmu about 600 y ago, and that Tin then branched into two varieties (Mal and Prai) some 200–300 y ago [6,32]. These time estimates are based on a calibration of the chronology of sound changes in Tin against reasonably secure datings of sound changes in neighboring languages; the actual time depth may be underestimated, but most likely by not more than a few centuries. Thus, the linguistic evidence would date the origin of the Mlabri at less than 1,000 y ago, in excellent agreement with the genetic evidence.

Other data that may shed light on the origins of the Mlabri, such as historical information, are scarce, since the Mlabri do not have a written language and the first recorded contact was only in 1936. However, the Tin Prai have an oral tradition concerning the origin of the Mlabri (J. Rischel, personal communication), in which several hundred years ago, Tin Prai villagers expelled two children and sent them downriver on a raft. They survived and escaped into the forest, turning to a foraging lifestyle and thus becoming the Mlabri. Although it is difficult to know how to evaluate such oral traditions, this story nevertheless intriguingly parallels the genetic and linguistic evidence concerning the origins of the Mlabri.

In sum, genetic, linguistic, and cultural data all suggest a founding event in the Mlabri, involving a single maternal lineage and 1–4 paternal lineages some 500–1,000 y ago, from an ancestral agricultural population. The Mlabri then subsequently adopted their present hunting and gathering lifestyle, possibly because the group size at the time of founding was too small to support an agricultural lifestyle. Other examples of such cultural reversion are rare; probably the best known involves Polynesian hunter–gatherers on the Chatham Islands and the South Island of New Zealand [33], who abandoned agriculture and adopted a maritime-based foraging subsistence because of the rich marine resources and the inability of these islands to support cultivation of tropical crops. Other hypothesized examples of cultural reversion, such as the Punan of Borneo [34], the Guajá and other lowland Amazonian groups [35], and the Sirionó of Bolivia [36], are controversial, as it is not clear whether these groups are descended directly from earlier hunter–gatherer groups or whether they indeed have undergone cultural reversion. Detailed genetic analyses, as carried out here for the Mlabri, may shed further light in these cases.

In any event, our conclusion that the Mlabri, a present-day group of hunters and gatherers, was founded recently and in all probability from an agricultural group further supports the contention that contemporary hunter–gatherer groups cannot be automatically assumed to represent the pre-agricultural lifestyle of human populations, descended unchanged from the Stone Age [37]. Indeed, even if they have not reverted from an agricultural lifestyle, most (if not all) contemporary hunter–gatherer groups interact with, and have evolved and changed along with, agricultural groups [38]. The Mlabri provide a unique opportunity to investigate the circumstances and consequences of a reversion from an agricultural to a hunting–gathering lifestyle that apparently was not dictated by purely ecological reasons (as in the case of Polynesian hunter–gatherers).

## Materials and Methods

### 

#### Samples

There are three linguistically distinct subgroups of Mlabri [39], designated A, B, and C. Subgroup A (also known erroneously as “Mrabri”) is the only group that has been studied in detail [1]; subgroup B (minor Mlabri) is practically extinct [2], and subgroup C (formerly “Yumbri”) comprises less than 30 people [39]. Blood samples and genealogies of 91 Mlabri (all from subgroup A) were obtained with informed consent in 1999, and cell lines were prepared and DNA was extracted from the cell lines. The genealogical data were used to identify and exclude known relatives from the genetic analyses. Data on mtDNA and Y-STR variation from six agricultural hill tribes in the same geographic region (Akha, Lahu, White Karen, Red Karen, CR Lisu [from near Chiang Rai], and MHS Lisu [from near Mae Hong Son]), all of whom speak Sino-Tibetan languages, were published previously [26].

#### Genetic analyses

The first hypervariable segment (HV1) of the mtDNA control region (nucleotide positions 16,024–16,385) was amplified and sequenced directly, as described previously [29], from 58 Mlabri. PCR analysis of the intergenic region between the cytochrome oxidase subunit II and lysine tRNA genes, which harbors an informative 9-bp deletion, was carried out as described previously [28]. Nine Y-STR loci (DYS385a, DYS385b, DYS389I, DYS389II, DYS390, DYS391, DYS392, DYS393, and DYS394) were amplified and genotypes determined, using previously described methods [30], for 54 Mlabri. Nine autosomal STR loci (D3S1358, vWA, FGA, D8S1179, D21S11, D18S51, D5S818, D13S317, and D7S820) plus the amelogenin locus were amplified with the AmpFLSTR Profiler Plus PCR Amplification Kit (Applied Biosystems, Foster City, California, United States), using 2–4 ng of DNA in a 15-μl reaction volume. Genotypes were determined by fragment analysis on an ABI377 (Applied Biosystems) for 35 Mlabri, 29 Lahu, and 30 individuals from each of the other hill tribes.

#### Statistical analyses

Genetic diversity, heterozygosity, and tests for goodness of fit to Hardy–Weinberg expectations were calculated with Arlequin 2.000 [40]. LD was estimated as the probability of the likelihood of the data assuming linkage equilibrium versus the likelihood of the data assuming association [19]; Arlequin 2.000 was used to obtain maximum-likelihood estimates of the haplotype frequencies for each pair of loci with the EM algorithm [41], and the null distribution of the *p*-value of the likelihood ratio test was generated by 10,000 random permutations. The program Bottleneck (http://www.ensam.inra.fr/URLB/bottleneck/bottleneck.html) was used to compare the observed heterozygosity at each autosomal STR locus to that expected at mutation–drift equilibrium for the observed number of alleles, assuming a stepwise mutation model. Bayesian-based coalescence analyses of Y-STR haplotypes [42] were performed using the software Batwing (http://www.maths.abdn.ac.uk/~ijw/downloads/batwing/batguide/node6.html) and previously described prior distributions for the initial effective population size, population growth rate, and Y-STR mutation rates [30]. The coalescence time for mtDNA HV1 sequences was also estimated by a Bayesian procedure [21] as described previously for Xq13.3 sequences [43], using the same initial effective population size and population growth rate priors as for the Y-STR analysis, and a γ-distribution with parameters α = 14.74 and β = 0.0005 (corresponding mean = 0.00737) as a prior for the mutation rate [44]. Resampling of mtDNA and Y-STR types, in order to estimate the magnitude of population size reduction needed to eliminate mtDNA and reduce Y-STR diversity, was performed with the software Resample (http://www.pbs.port.ac.uk/~woodm/resample.htm). Bayesian analysis of the number of founders for the Mlabri was performed by pooling the mtDNA types in the other hill tribes to obtain a starting population, from which a certain number of founding mtDNA types were selected at random, assuming a uniform prior distribution between one and 20 founders. The sample was then allowed to grow from the number of founders to size 300 (the current size of the Mlabri population) over various time intervals, such that the shorter the time interval, the faster the growth rate. Simulations were performed both under the assumption of no new mutations, and with a mutation rate of one mutation/sequence/10,000 y. For each combination of parameters, 1,000,000 simulations were carried out. The simulation results were converted via Bayes's theorem into a posterior probability for the number of founding individuals, conditioned on the observation of no diversity in a random sample of size 58 (the sample size in this study). In practice, the posterior probability distributions were independent of the mutation rate (analyses not shown).

## Abbreviations

LD: linkage disequilibrium
STR: short tandem repeat
Y-STR: Y-chromosome short tandem repeat
